# Unlocking the ferroptotic window: Lipidomic rewiring and metabolic addiction in EMT-driven breast cancer resistance

**DOI:** 10.1038/s41420-026-03217-5

**Published:** 2026-07-04

**Authors:** Jian Lu, Chrysa Filippopoulou, Junjie Sun, Pengtao Hu, Chaoyue Pan, Xiaoyan Wu, Xidong Gu, Xiaohong Xie, Qijin Shu, Georgios Giamas

**Affiliations:** 1 International Oncology Institute, The First Affiliated Hospital of Zhejiang Chinese Medical University, Oncology Department of the First Affiliated Hospital of Zhejiang Chinese Medical University, Hangzhou, China; 2 Department of General Surgery, Nanjing First Hospital, Nanjing Medical University, Nanjing, China; 3 Department of Breast Surgery, The First Affiliated Hospital of Zhejiang Chinese Medical University, Hangzhou, China; 4 Department of Biochemistry and Biomedicine, School of Life Sciences, University of Sussex, Brighton, UK

**Keywords:** Breast cancer, Molecular biology

## Abstract

Breast cancer (BC) continues to pose a substantial clinical challenge due to acquired resistance induced by epithelial-mesenchymal transition (EMT). Nevertheless, this adaptive evolution, which frequently takes the form of a highly plastic, partial EMT (p-EMT) state, induces a profound lipidomic reconfiguration and iron dysregulation, thereby inadvertently revealing a targetable metabolic vulnerability: ferroptosis. In this Perspective, we outline the hierarchical molecular logic that governs this susceptibility, emphasizing the manner in which progressive p53 mutations (ranging from loss-of-function to gain-of-function) transform p-EMT cells from passive sensitization to an extreme “metabolic addiction.” We argue that conventional “occupancy-driven” kinase inhibitors are unable to eliminate these resistant populations because they are unable to dismantle the essential non-catalytic scaffolding functions of core EMT-induced kinases (EIKs). As a result, we suggest a paradigm shift toward a chemical biology approach that is “event-driven.” The p-EMT infrastructure can be irreversibly destroyed and resistant cells can be compelled to undergo catastrophic lipid peroxidation by deploying proteolysis targeting chimeras (PROTACs) against concealed scaffold super-hubs, particularly AXL and lemur tail kinase 3 (LMTK3), which are indispensable for stabilizing the hybrid p-EMT infrastructure. Additionally, we investigate the spatiotemporal modulation of this synthetic lethal axis by the tumor microenvironment (TME) through matrix mechanics and extracellular vesicles (EVs). Ultimately, we suggest a multimodal liquid biopsy strategy that couples specific oxidized phospholipid signatures with circulating tumor DNA (ctDNA) kinetics to precisely monitor in vivo ferroptotic events. This approach offers a transformative roadmap for eradicating minimal residual disease (MRD) and surmounting BC dormancy.

## Introduction

Acquired drug resistance continues to pose an insurmountable obstacle in clinical treatment, despite the substantial progress made in precision medicine for breast cancer (BC) in recent years [1–4]. Tumor cells frequently endure adaptive evolution under therapeutic pressure, resulting in disease recurrence, regardless of whether this occurs during endocrine therapy for hormone receptor-positive (HR+ ) patients or conventional chemotherapy for triple-negative breast cancer (TNBC) [5–7]. Epithelial-mesenchymal transition (EMT) has been implicated as a fundamental driver in this dynamic process of resistance evolution [8]. The significant enrichment of mesenchymal markers in circulating tumor cells (CTCs) and disseminated tumor cells (DTCs) after traditional systemic therapies, which is strongly correlated with multi-drug resistance (MDR), minimal residual disease (MRD), and a poor prognosis for patients, is clinical evidence of this adaptive evolution [9, 10]. However, there is a large “therapeutic void” in modern oncology: although standard-of-care treatments successfully reduce the size of the epithelial-like tumor mass, they often fail to eradicate these highly malleable p-EMT populations because there are no targeted agents that can take advantage of their particular survival vulnerabilities [11, 12]. This inability to empty the “p-EMT reservoir” frequently creates the conditions for the disease’s unavoidable return [13]. BC cells not only acquire improved migratory and invasive capabilities through EMT, but also alter their survival strategies, thereby evading apoptotic signals and establishing a drug-resistant, resilient population [14].

It is now widely acknowledged that EMT is not a binary switch, but rather a fluid continuum. Crucially, when BC cells evade therapy, they frequently exist in a hybrid partial EMT (p-EMT) state [15, 16]. This metastable phenotype enables cells to maintain epithelial characteristics, including cell-cell adhesion, which are fundamental for collective migration and survival, while simultaneously acquiring mesenchymal-like metabolic traits [17]. This high-plasticity state, rather than a complete phenotypic revision, we contend, serves as the primary reservoir for drug-tolerant persisters (DTPs) and establishes a distinctive, targetable window for ferroptosis [18, 19].

In order to comprehend the manner in which EMT exposes cells to ferroptosis, it is necessary to first examine the fundamental regulation of this mechanism of cell death [20, 21]. Traditionally, the tumor suppressor p53 functions as a master regulator of metabolic stress, frequently mitigating ferroptosis by transcriptionally regulating antioxidant targets such as SLC7A11 [22]. However, the mutation status of p53 fundamentally rewires this basal defense during the adaptive evolution of BC, resulting in distinct vulnerability patterns [23, 24].

Rather than establishing a singular linear progression axis, we propose that the susceptibility of BC cells to ferroptosis is determined by a hierarchical molecular logic that is closely linked to the p53 status. Initially, the Basal Defense State is illustrated by wild-type (WT) p53 models, such as MCF7. In these cells, p53 functions as a “metabolic shield” by transcriptionally sustaining antioxidant targets (e.g., SLC7A11), thereby ensuring a high level of ferroptosis tolerance [25].

Secondly, the Passive Sensitization pattern is observed in models such as T47D, where p53 loss-of-function (LOF) mutations dismantle this “antioxidant brake“ [26]. These cells become passively susceptible to extrinsic oxidative stress when their foundational transcriptional shield is removed.

Third, mesenchymal-like TNBC models, such as MDA-MB-231, exhibit a more aggressive Active Sensitization pattern. In these contexts, p53 gain-of-function (GOF) mutations are not merely defensive deficiencies; they are the “driving engines” of metabolic re-engineering. The accumulation of Reactive Oxygen Species (ROS) and the synthesis of lipids are actively stimulated by mutant p53, resulting in an extreme compensatory reliance on Glutathione Peroxidase 4 (GPX4), a condition known as Metabolic Addiction [27]. Consequently, the transition from WT to LOF, and subsequently to GOF, signifies a qualitative escalation from “robust defense” to “compromised barrier” and, ultimately, to “catastrophic addiction.” While these classic cell lines represent different static metabolic snapshots in BC heterogeneity, changes in p53 state are often accompanied by more complex dynamic network remodeling in the clonal evolution of clinical patients.

Importantly, during clonal evolution under therapeutic pressure, the activation of the EMT program is often linked to the development of such aggressive p53 GOF phenotypes [28]. Because of this chemical synergy, mutant p53 proteins can work with key EMT transcription factors, most notably ZEB1, to systematically remodel the cellular lipidome in addition to promoting morphological plasticity [29]. Therefore, in order to avoid treatment, BC cells undergoing EMT do more than just alter their appearance; they incorporate these carcinogenic signals to orchestrate significant metabolic changes, particularly the enrichment of polyunsaturated fatty acids (PUFAs), which eventually creates a targetable vulnerability window to ferroptosis [30]. According to this paradigm, mesenchymal-like cells’ sensitivity represents a basic trade-off of their adaptive evolution rather than a chance result [19].

Both clinical transcriptomic datasets and cell line panels provide evidence that the transition from p53 LOF to GOF status signifies a qualitative shift from “compromised defense” to “metabolic addiction.” Across the spectrum of BC heterogeneity, this shift fundamentally determines the ferroptosis susceptibility landscape [23].

New evidence is increasingly indicating that BC cells that are undergoing EMT to acquire drug resistance frequently exhibit substantial lipid metabolic remodeling, which has the potential to create a specific susceptibility window to ferroptosis [19]. Classical ferroptosis inducers (e.g., Erastin or RSL3) are currently the primary focus of emerging therapeutic strategies that aim to exploit this vulnerability. These strategies involve combining conventional ATP-competitive kinase inhibitors that target EMT-associated receptors with FINs [31].

Nevertheless, the mere identification of this Achilles’ heel is insufficient to translate it into a clinical cure. This paper offers a fundamental critique: the current state of drug development that targets the EMT-ferroptosis axis is excessively preoccupied with conventional ATP-competitive kinase inhibitors. This “occupancy-driven” pharmacology strategy contains a fatal blind spot.

In this perspective, we utilize the AXL receptor tyrosine kinase as a representative proof-of-concept paradigm to illustrate that many core kinases not only function catalytically but also serve as essential scaffold proteins in the complex EMT network remodeling, mediating complex protein-protein interactions (PPIs) [32]. For example, these kinase scaffolds physically tether cytoskeletal elements and connect extracellular microenvironmental stimuli to downstream metabolic transcription factors (such as Sterol regulatory element-binding protein 1 (SREBP1)), in addition to their enzymatic activity. Consequently, the cells are confined to a mesenchymal and lipid-addicted state [33]. Although conventional inhibitors can obstruct kinase phosphorylation cascades, they are unable to disassemble these physical scaffolds. This enables cancer cells to easily compensate by bypassing signaling pathways, thereby escaping and maintaining drug resistance. The use of targeted protein degradation (TPD), such as proteolysis targeting chimeras (PROTACs), in the EMT-ferroptosis axis is still mainly unexplored, despite the fact that TPD has quickly transformed treatment paradigms for other cancers by completely degrading disease-causing proteins [34].

We suggest that future therapeutic improvements should investigate a shift toward “event-driven” techniques, such as TPD, typified by PROTACs, as conventional inhibitors frequently preserve non-catalytic scaffolding roles. We speculate that this kind of strategy might be quite successful in exploiting this synthetic lethal axis. This represents not merely an incremental improvement in intervention tools, but a fundamental dismantling of the robust networks that sustain drug resistance. We can completely “erase” the scaffold proteins that drive EMT by inducing the formation of ternary complexes of target proteins and hijacking the ubiquitin-proteasome system. The lipid metabolic compensation mechanisms of BC cells are irreversibly dismantled by this physical eradication, thereby breaching their antioxidant defenses and driving them toward fatal lipid peroxidation.

## Molecular Mechanisms: Rewiring of Metabolism and Signaling during BC EMT

EMT is not merely a physical transformation from “square” to “spiky” cell morphology that occurs during the progression of BC; it also represents a significant restructuring of metabolic and signaling pathways [35]. This remodeling simultaneously facilitates ferroptosis and provides survival advantages to mesenchymal-like cells [35, 36].

### Metabolic Reprogramming: Synergistic Lipid Sensitization and Iron Dysregulation

The phenotypic shift from an epithelial to a mesenchymal state is fundamentally accompanied by profound changes in cellular lipid composition [19]. The most notable characteristic of this transition is the significant upregulation of LPCAT3 and ACSL4 in BC cells residing in the p-EMT state. Canonical EMT transcription factors, including ZEB1, Snail, and Twist, directly determine the fate of cellular lipids in addition to their role in driving morphological alterations [37]. These master regulators regulate lipid absorption, lipogenesis, and ultimate peroxidation vulnerability by employing ACSL4 and LPCAT3 as vital effectors [38]. Interestingly, ZEB1 has become a key player in lipid modification, which makes cells more susceptible to ferroptosis [29]. Together with effector enzymes like ACSL4 and LPCAT3, it facilitates the effective integration of polyunsaturated fatty acids (PUFAs) into membrane phospholipids, resulting in a significant increase in the proportion of PUFA-PLs on the cell membrane [39–41].

This modification in the composition of membrane lipids essentially predisposes cells to extreme sensitivity to lipid peroxidation, establishing the substrate basis for ferroptosis from the perspective of cellular fate [42, 43]. The bis-allylic carbons situated between two carbon-carbon double bonds in PUFA chains exhibit an exceptionally low carbon-hydrogen bond dissociation energy (BDE) from a chemical thermodynamic and kinetic perspective [43, 44]. This distinctive chemical composition renders them highly susceptible to hydrogen abstraction assaults by ROS, particularly hydroxyl radicals, which are produced by microenvironmental or organelle stress. As a result, they function as an “ideal tinder” for the initiation of catastrophic radical chain oxidation reactions [42, 44, 45]. Therefore, mesenchymal-like cells undergo “sensitization” of their membrane components to enhance their invasive capabilities and survival. These mesenchymal cells develop a deep metabolic dependence on classical ferroptosis defensive mechanisms in order to survive the dangerous buildup of highly oxidizable PUFA-PLs. This mainly entails an over-reliance on the parallel ferroptosis suppressor protein 1 (FSP1)/CoQ10 pathway and the system Xc- (SLC7A11)/glutathione (GSH)/GPX4 antioxidant axis [46]. The final threshold for ferroptosis is determined by the dynamic interaction between compensatory hyperactivation of these antioxidant defense lines and ZEB1-driven lipid sensitization [19]. As a result, this lipidomic rewiring linked to EMT subtly pushes cells in the direction of a precarious edge where they are well positioned for targeted ferroptotic initiation.

It is critically important to note that the initiation of catastrophic lipid peroxidation necessitates a biochemical catalyst, despite the fact that the accumulation of PUFA-PLs serves as the highly combustible “tinder” for ferroptosis. Importantly, the p-EMT program that promotes lipid rewiring also coordinates a distinct metabolic reprogramming in iron homeostasis, and thus the supply of this catalyst is not a singular occurrence [19, 47]. Such hybrid cells usually drastically increase the expression of TFRC to meet their needs for rapid metabolic turnover, which greatly enhances their ability to obtain extracellular iron [47, 48]. At the same time, ferritin, the main intracellular iron storage protein, experiences similar changes in its expression or breakdown patterns. The downregulation of iron storage capacity further reduces the cell’s ability to sequester and buffer free iron [19, 49].

This characteristic and extreme metabolic pattern of “high intake, low storage” directly and significantly expands the intracellular labile iron pool (LIP) [19, 49]. The accumulation of free iron, which serves as a potent biochemical catalyst, substantially accelerates the Fenton reaction [50, 51]. The cell’s original redox homeostasis is not only disrupted by this imbalance, but it also provides the critical propelling force for lipid peroxidation and the subsequent iron-induced death cascade.

The master EMT transcription factor ZEB1 is located at the intersection of these parallel pathways, which functions as a comprehensive metabolic rheostat, coordinating both the iron dysregulation and the lipid sensitization. It is vital to note that in TNBC models, this process is stimulated by p53 GOF mutations, which form a transcriptional complex with ZEB1 to synergistically hyperactivate the lipogenic program [19]. ZEB1 functions as a metabolic rheostat that directly stimulates the absorption and mobilization of lipids in addition to its traditional function of suppressing epithelial indicators. It has been demonstrated that ZEB1 specifically drives the development of enzymes involved in the metabolism of polyunsaturated fatty acids (PUFAs), accumulating the “combustible” substrates required for the execution of ferroptosis [29]. Because of its location, ZEB1 serves as a crucial link between the mesenchymal state and strong ferroptotic priming [52].

### The EIK Network: Deciphering the Scaffolding Infrastructure of Metabolic Addiction

In this dynamic evolutionary process, core kinase promoters of EMT in BC are crucial [53]. Nevertheless, the continuous improvement of chemical biology tools has resulted in a shift in the focus of researchers from broad classical kinase cascades (such as phosphoinositide 3-kinase/protein kinase B (PI3K/Akt) and mitogen-activated protein kinase (MAPK)) to “bridge kinases” that specifically mediate environmental stress, drive drug-tolerant persisters (DTP), and profoundly reshape ferroptosis defense networks [54, 55].

It is imperative to acknowledge that AXL is merely the top of the iceberg in the EIK network. This network includes not only well-characterized RTKs but also deep intracellular scaffold kinases, including p21-activated kinase 1 (PAK1) and lemur tail kinase 3 (LMTK3) [16, 56]. These centers collectively orchestrate the spatiotemporal maintenance of the p-EMT state, thereby rewiring lipid metabolism and ferroptosis defense networks. Among these, the AXL receptor tyrosine kinase has demonstrated exceptional potential as a therapeutic intervention target and offers the most clinically advanced example of this multidimensional regulation [57, 58]. AXL is constitutively expressed in epithelial BC cells as a central member of the Tyro3, Axl and Mer (TAM) kinase family. However, it undergoes significant activation and upregulation during EMT that is induced by targeted therapy or chemotherapy stress [59, 60]. The cytoskeleton is directly remodeled to increase invasiveness by the abnormal activation of AXL, which also profoundly rewrites the cellular lipid fate as a fundamental “commander” of transcription and metabolism [61–64].

In particular, the AXL-driven stromalization network demonstrates significant synergistic lethality in BC cells in conjunction with lipid remodeling [57, 65]. AXL, a membrane receptor kinase, does not directly modify metabolic enzymes; rather, it reshapes the cellular transcriptional landscape through complex signaling networks [65]. The downstream PI3K/Akt/mTOR and Hippo/YAP cascades of AXL converge on SREBP1, where it functions as a primary transcriptional rheostat [66–68]. This axis effectively stimulates the expression of ferroptosis-executing enzymes (ACSL4 and LPCAT3), thereby establishing a combustible lipid environment within drug-resistant cells [19, 69, 70].

This cascade reaction results in the highly efficient enrichment of PUFAs in membrane phospholipids, thereby creating an extremely combustible lipid environment at the physicochemical level within drug-resistant cells that is highly susceptible to initiating radical chain reactions [35, 69]. Conversely, this membrane lipid remodeling for invasiveness induces a metabolic compromise in highly plastic p-EMT cells [71, 72]. In order to mitigate membrane lipid instability, AXL-overexpressing cells develop a fatal “addiction” to antioxidant defense systems such as GPX4 or FSP1 [19, 46, 57]. This implies that AXL serves as a critical compensatory survival mechanism to prevent the catastrophic lipid peroxidation that is inherent to the mesenchymal state, while also enabling cells to evade classical apoptosis. If this fragile antioxidant synergy is disrupted, cells will swiftly enter a catastrophic lipid peroxidation crisis.

This “AXL-lipid remodeling-ferroptosis susceptibility” axis offers an exceptionally distinct molecular target for overcoming BC drug resistance from the perspective of chemical biology-based drug design. In recent years, structure-based drug design (SBDD) has developed a series of small-molecule kinase inhibitors that are highly selective and target the ATP-binding pocket of AXL, such as bemcentinib [57, 73, 74]. Blocking the AXL signaling pathway with these highly specific chemical probes or inhibitors not only effectively reverses the EMT phenotype but also deprives drug-resistant clones of the fundamental drivers maintaining lipid homeostasis [74, 75]. The foundation of BC recurrence is eliminated by the unprecedented synergistic lethal effects that are unleashed in targeted drug-resistant persistent cells when further combined with ferroptosis inducers.

The theoretical feasibility of our proposed paradigm is further supported by the efficacy of targeting the AXL-lipid axis [19]. Consequently, rather than being a single-receptor cascade, the EMT-ferroptosis axis should be understood as a multi-layered kinase network [76]. The robust, redundant infrastructure that supports drug-resistant clones is provided by the synergy between surface-level receptor tyrosine kinases (RTKs) (like AXL) and deep intracellular scaffolders (like LMTK3 and PAK1) [77]. Therefore, it is mechanistically necessary to physically dismantle this entire network via event-driven techniques [78]. However, pipeline hazards are present when the scope of intervention is limited to RTKs [79].

The EMT in BC is a dynamic process that is intricately linked to a variety of biochemical signals and mechanical forces. Consequently, the scope of chemical biology must be expanded to encompass the more complex intracellular serine/threonine (Ser/Thr) kinase networks. For example, LMTK3 and other complex dual-specificity kinases serve as both scaffolding and catalytic elements in anchoring cells within a high-plasticity p-EMT state, which is defined by the simultaneous expression of epithelial (e.g., E-cadherin) and mesenchymal (e.g., vimentin) markers. These roles are covert yet critical. This abnormal “arrest” in a metastable state indirectly reshapes lipid metabolism profiles and alters the physical invasiveness of cells [80–89]. Cytoskeletal reorganization is frequently profoundly influenced by these kinases. Their abnormal expression not only alters the physical invasiveness of cells but also indirectly alters the lipid metabolism profiles by modulating the nuclear translocation of critical transcription factors [56, 80, 81, 90]. Furthermore, the substantial non-catalytic domains of these kinases may function as physical platforms for the “metabolic machinery” that is present within tissue cells [91]. For instance, LMTK3 and AXL can be recruited to the contact sites between the endoplasmic reticulum (ER) and lipid droplets (LDs). Through physical interactions with lipid droplet-associated proteins, such as the perilipin family, they assist in the isolation of free sensitive lipids (such as polyunsaturated fatty acids, PUFAs) into highly stable lipid droplet reservoirs [92, 93]. This scaffold function is not only a physical cornerstone for cells to maintain metabolic defense barriers under duress, but also a bridge for signal transduction [94, 95].

Identifying effective active sites is a challenge for traditional high-throughput screening methods, as these kinases contain extensive non-catalytic domains. This provides an optimal, untapped chemical space for the discovery of cryptic pockets through activity-based proteomics (ABPP) and the development of PROTAC degradation strategies. The potential to disrupt EMT processes at a more fundamental skeletal and metabolic intersection point is demonstrated by the targeting of these deep intracellular scaffold kinases.

The literature currently in publication offers complex perspectives on AXL’s function in this situation. Numerous studies highlight AXL as a crucial ferroptosis inhibitor that gives drug-resistant cells a survival advantage, despite some models suggesting that its activation is linked to sensitivity [96–101]. We suggest that AXL functions as a vital “defense barrier” to prevent catastrophic lipid peroxidation in mesenchymal cells, which would otherwise be doomed by ZEB1-driven lipid reprogramming, rather than immediately initiating the ferroptosis cascade. This reliance results in a special “metabolic addiction,” in which the cell’s antioxidant defense mechanisms must be destroyed by physical removal of AXL (instead of merely catalytic inhibition) in order to unleash a combinatorial fatal effect.

## TME and Spatiotemporal Regulation in BC

The EMT-ferroptosis axis is dynamically regulated by the complex TME during the progression of BC, rather than operating independently within individual cells [68, 102]. The scope of chemical biology research is broadening to encompass the physical microenvironment, intercellular communication networks, and intracellular processes, thereby revealing the spatial propagation mechanisms of drug-resistant phenotypes [103, 104].

Beyond functioning as passive conduits for intercellular communication, EVs within the TME mediate a critical evolutionary trade-off [19, 105]. On the one hand, drug-resistant p-EMT cells “package” core oncogenic kinases (e.g., AXL) and specific miRNAs within EVs, thereby delivering them to adjacent epithelial-like cells. This effectively induces the epithelial-to-mesenchymal transition (EMT) and confers resistance to chemotherapeutic agents, thereby facilitating the spatial expansion of drug-resistant phenotypes [105, 106]. Nevertheless, the acquisition of this survival advantage is associated with a substantial metabolic cost. EVs inadvertently introduce lipid components that are abundant in PUFAs and lipid remodeling enzymes (e.g., ACSL4) into the membrane structures of recipient cells while transmitting oncogenic signals [19, 103]. While this “bundled” metabolic remodeling enhances invasiveness, it simultaneously compromises membrane chemical stability. It converts cells that were previously resistant to oxidative stress into “ferroptosis targets” that are highly susceptible. Therefore, EVs provide an ideal synthetic lethality entry point for spatially “encircling” drug-resistant clones using ferroptosis inducers, essentially trading metabolic safety for survival at the microenvironmental scale.

Cellular metabolic fate is significantly influenced by the dense stroma that is a hallmark of BC, as a result of physical mechanical forces. In this microenvironmental interaction, high matrix stiffness functions as a critical mechanical signal that efficiently activates mechanosensitive pathways, including YAP/TAZ [107, 108]. In addition to their role as potent inducers of EMT, these pathways also directly involve themselves in the metabolic regulatory networks that regulate amino acid and lipid transport [108, 109]. Simultaneously, cancer cells undergo significant endoplasmic reticulum (ER) stress as a result of sustained microenvironmental mechanical stress [110]. This intracellular ROS production level is jointly and precisely regulated by the EMT signaling cascade, which is interconnected with the organelle stress state [110, 111].

BC cells demonstrate intricate metabolic adaptability as a result of the pervasive crosstalk between mechanical signals and organelle stress [68, 110]. Under high mechanical stress, cells compensatorily upregulate antioxidant defense systems such as FSP1 or GPX4 to mitigate potential oxidative damage induced by physical stress through the synergistic action of YAP/TAZ-mediated transcriptional regulation and endoplasmic reticulum stress [46, 112]. This molecular network remodeling, which is driven by the tissue-physiological niche, dynamically modulates the lipid peroxidation threshold of BC cells, thereby fundamentally determining their resistance to ferroptosis intervention within highly heterogeneous and complex physical microenvironments.

The spatial regulation of the EMT-ferroptosis axis is intricately linked to other essential TME elements, such as hypoxia, angiogenesis, and immune cell infiltration, in addition to EVs and mechanical stresses [68, 103, 113]. Dense BC stroma is characterized by hypoxia, which drives lipid droplet accumulation, which sequesters PUFAs and temporarily protects p-EMT cells from lipid peroxidation while also strongly triggering EMT via Hypoxia-inducible factor 1-alpha (HIF-1α) activation [16, 114]. We think that this “lipid isolation” is an active survival strategy powered by the EIKs scaffold network rather than a passive outcome of hypoxia-induced deterioration. Scaffold proteins like LMTK3/AXL offer physical anchors that are necessary for the stability and activation of HIF-1α under hypoxic stress. In order to quickly transfer readily oxidized PUFAs into lipid droplets for “strategic concealment,” these scaffold proteins work in concert to construct lipid transport complexes. The primary defense mechanism of p-EMT cells under severe oxidative stress is this physical isolation, which is reinforced by scaffold proteins. Therefore, this physical barrier may not be broken by merely blocking their enzymatic activity; this defense platform may only be completely demolished by complete protein disintegration.

In addition to aggravating this hypoxic gradient, defective tumor angiogenesis also specifically modifies local iron availability, requiring BC cells to dynamically modify their TFRC and ferritin levels in order to survive [115]. Moreover, the immunological microenvironment has two functions. Tumor-associated macrophages (TAMs), which are frequently polarized toward a pro-tumorigenic state by EMT-derived signals (including particular EIKs like LMTK3), release cytokines that either enhance antioxidant defenses or, in response to particular therapeutic pressures, release lipid mediators that work in concert with ferroptosis inducers [56, 85, 116]. This complex, multi-layered interaction between immune cells, ECM remodeling, and oxygen/nutrient deprivation, which together determine the ferroptotic threshold of resistant clones in vivo [19], must be taken into consideration in a thorough understanding of the TME.

## The Chemical Biology Toolbox: Multi-omics-Driven Discoveries and Small Molecule Interventions

Chemical biology provides a potent set of precision instruments to elucidate and intervene in the complex EMT-ferroptosis axis in BC. Utilizing small-molecule probes and multidimensional omics technologies, it is possible to penetrate the microscopic protein active sites from the macroscopic tissue landscape, thereby facilitating targeted attacks against drug resistance vulnerabilities.

### Multi-omics Integration and Chemical Probes: Mapping the Dynamic Panorama of Drug Resistance Evolution

Conventional transcriptomics and proteomics often fail to capture the dynamic, functional states of kinases and real-time lipid flux. To bridge this mechanistic gap, chemical proteomics provides an essential instrument. Chemical proteomics provides an additional potent instrument for disentangling this intricate drug resistance network at the protein functional level [51, 117]. Researchers can employ functional group probes to identify active kinase pockets and protein groups that are critical to mesenchymal BC within large-scale kinase panels using ABPP technology [117, 118].

For example, researchers can employ click chemistry in conjunction with custom lipid probes that contain alkyne tags to not only monitor the subcellular localization of sensitized lipids but also directly capture effector protein complexes that undergo covalent cross-linking with peroxidized lipids during the execution phase of ferroptosis in situ within living cells [119]. One of the most traditional chemical strategies for resolving PUFA-PL enrichment networks is the design and synthesis of bioorthogonal assays that contain terminal alkyne groups, such as 19-Alkyne arachidonic acid (Alk-AA), in order to monitor lipid remodeling [120].

By introducing Alk-AA into BC cells that are undergoing EMT, the probe can be recognized by ACSL4 and LPCAT3 and incorporated into the phospholipids of the cell membrane [120, 121]. Consequently, researchers may apply copper-catalyzed azide-alkyne cycloaddition (CuAAC, a type of click chemistry) to affix fluorescent groups for subcellular high-resolution imaging or biotin markers for enrichment and mass spectrometry identification [119, 122].

Furthermore, fluorescent or mass spectrometry probes based on diphenylphosphine derivatives have been devised to detect lipid peroxides (L-OOH) during the execution phase of ferroptosis. These probes utilize the chemical reactivity of hydrogen peroxides to specifically reduce them into hydroxyl substrates [123], thereby facilitating the in situ, quantitative chemical capture of lipid fragility in mesenchymal BC cells [72, 124]. This innovative method not only accurately identifies specific metabolic enzymes or kinases that are highly activated during EMT, but it also surpasses the constraints of conventional structural biology to reveal enigmatic druggable pockets. This establishes a strong foundation for the creation of innovative pharmaceuticals that are “first-in-class” and specifically target this synthetic lethal axis.

### Precision Targeting with Small-Molecule Inhibitors and Degraders: From Druggable Pockets to Reshaping Protein Homeostasis

The ultimate objective of chemical biology is to convert the lipid vulnerability of BC into actionable therapeutic strategies [63]. The precision of pharmacophore design is illustrated by the development of highly selective small molecules that target core bridge kinases like AXL within the framework of SBDD, such as bemcentinib (R428) [57, 125]. The ATP-binding cleft of AXL is occupied by bemcentinib with nanomolar affinity. Its aminotriazole moiety forms a robust network of double hydrogen bonds with key amino acid residues in the kinase hinge region, thereby specifically blocking AXL autophosphorylation and its drive of downstream EMT signaling [59, 60, 126].

It is important to note that the catalytic activity of kinases alone is frequently insufficient to completely reverse persistent drug resistance states [34, 127]. In recent years, PROTACs have emerged as disruptive chemical instruments in targeted cancer therapy [78]. In this perspective, we propose that the application of this degradation technology to critical scaffolding EIKs could offer a novel approach to the complete blockade of the EMT-ferroptosis axis [128]. This approach is especially relevant for p53 GOF-driven BC, as the “active sensitization” produced by mutant p53 necessitates the physical removal of EIK scaffolds in order to collapse the robust antioxidant defense networks.

Currently, therapeutic strategies that specifically employ PROTACs to exploit the EMT-ferroptosis axis are primarily in the preclinical development stage, despite the fact that PROTACs targeting canonical oncogenic drivers (such as the estrogen or androgen receptors) have successfully advanced into clinical trials [129–131]. Although there are currently no PROTAC compounds that explicitly target the EMT-ferroptosis metabolic axis in clinical trials, we view this idea as a central potential proposition in this perspective. Nevertheless, this is a landscape that is undergoing a swift transformation. The profound clinical relevance of this approach is underscored by the successful in vitro and in vivo validation and active development of next-generation AXL-targeted degraders [34].

A critical and highly anticipated frontier in the overcoming of refractory BC is the translation of these ‘event-driven’ chemical tools from preclinical models to clinical applications. Moving forward, this is a critical area. AXL-PROTACs induce the formation of a ternary complex involving the target protein, resulting in the complete degradation of AXL via the ubiquitin-proteasome system, by connecting AXL’s binding ligand to the recruitment ligand of E3 ubiquitin ligases (e.g., cereblon, CRBN or von Hippel-Lindau, VHL) using flexible linkers such as polyethylene glycol (PEG) or alkyl chains [34, 132]. This approach not only eliminates AXL’s kinase activity but also disrupts its non-catalytic function as a scaffold protein in microenvironmental signaling.

Additionally, breakthroughs have been reached through chemical interventions that target ACSL4, the primary metabolic enzyme responsible for promoting membrane component sensitization [69, 133]. Initially, classic thiazolidinedione (TZD) compounds (e.g., rosiglitazone) were developed as peroxisome proliferator-activated receptor gamma (PPARγ) agonists. However, subsequent chemical proteomics disclosed their off-target inhibitory effects on ACSL4 [69]. Novel ACSL4-specific inhibitors (e.g., PRG349) were developed to block PUFA activation at its source by using structural optimization to remove the PPARγ-activating pharmacophore while retaining the inhibitory framework that targets the ACSL4 catalytic pocket [69, 134]. Creating an exceptionally tight synergistic lethal network within drug-resistant BC cells will be achieved by combining these lipid metabolism-targeting chemical tools with the kinase degraders (PROTACs) or traditional ferroptosis inducers (such as the covalent inhibitor RSL3 targeting GPX4).

It is imperative to underscore that a signaling pathway validation system that is exceedingly rigorous must be implemented to establish a connection between the reversal of actual biological phenotypes and target engagement in the chemical space, regardless of whether the development is of novel covalent inhibitors or PROTAC degradation agents [135]. Establishing a strict link between chemical target engagement and phenotypic reversal is still important in chemical biology [136]. It is crucial to accurately measure the target degradation efficiency and track the phosphorylation dynamics of downstream effectors (such as the PI3K/Akt or YAP/TAZ pathways) after using PROTACs that target scaffold kinases [137]. The fundamental requirement that guarantees the synthetic lethal method stays on course in preclinical settings is this exact biochemical confirmation [138].

### Perspective: Redefining Druggability—Expanding the Target Intervention Landscape of the Hidden Scaffold Kinase Network

Limiting “event-driven” degradation strategies to AXL alone is demonstrably inadequate. Numerous kinases are concealed within the extensive network of EMT in BC. These kinases are critical to tumorigenesis and phenotypic transformation, but they have been traditionally considered “undruggable” due to the absence of typical catalytic domains. For example, certain intracellular or transmembrane kinases that possess intricate non-catalytic functions frequently depend on their extensive domains to serve as super-hubs for signal transduction.

For instance, members of the p21-activated kinase (PAK) family, particularly PAK1, are frequently amplified or overexpressed in BC and drive EMT through both kinase-dependent and kinase-independent scaffolding functions. As a central node, PAK1 can simultaneously coordinate cytoskeletal remodeling and activate the Raf/MEK/ERK pathway. A conventional PAK1 ATP-competitive inhibitor might block its catalytic activity toward downstream effectors, but the physical scaffold would remain, continuing to tether signaling complexes and maintain a mesenchymal phenotype. A PAK1-targeting PROTAC, however, could eliminate the entire protein, dismantling this physical platform for signal integration and thereby collapsing the pro-EMT network more completely [139, 140].

Similarly, the non-receptor tyrosine kinase SRC, while possessing a well-defined catalytic domain, also utilizes its SH2 and SH3 domains to scaffold multi-protein complexes at focal adhesions. This scaffolding function is critical for transducing mechanical signals from the stiffened TME, a key driver of EMT and metabolic reprogramming. Degrading SRC would not only block its kinase activity but also disrupt these vital adhesive and mechanosignaling complexes, potentially synergizing with therapies targeting matrix stiffness [141, 142].

We believe that creating new PROTACs to target these hidden scaffold super-hubs, especially LMTK3 and PAK1, is a promising future path for chemical biology. We speculate that applying the “event-driven” degradation model to these deep intracellular targets could significantly alter the druggability landscape by extending the concepts shown with AXL. By extending the “event-driven” degradation paradigm to these deep intracellular targets, we can dismantle the physical substrates that tether p-EMT signaling to metabolic addiction. By degrading LMTK3, the entire pro-p-EMT platform collapses, fundamentally rewiring the druggability landscape of treatment-resistant BC.

## Translational Challenges and Future Perspectives

In order to transfer the synthetic lethality concept of “EMT-ferroptosis” from the laboratory to the clinical setting, it is necessary to surmount numerous translational obstacles. This process necessitates not only the development of novel drugs but also the acquisition of profound insights into the dynamic evolution of tumors.

### Overcoming BC Drug Resistance: Combination Therapy to Eliminate MRD

The greatest potential for clinical translation is possessed by small-molecule drugs that target the EMT-ferroptosis axis through profound synergistic application with existing standard therapies [47]. The mechanistic justification for this particular combination is derived from the observation that the compensatory hyperactivation of specific EIKs, such as AXL and LMTK3, is frequently induced by protracted endocrine therapeutic pressure in HR + BC cells [143]. By establishing alternative survival networks and promoting a robust EMT phenotype [85], these scaffolding kinases enable cancer cells to circumvent estrogen receptor blockade. Paradoxically, as delineated in our proposed model, this EIK-driven resistance mechanism fundamentally rewires the lipidome, resulting in a profound, targetable dependency on antioxidant systems [144, 145]. Therefore, combining ferroptosis inducers or novel small-molecule inhibitors that target EIKs with conventional agents such as tamoxifen or fulvestrant can efficiently disrupt the metabolic homeostasis of tumor cells in the clinical treatment of HR + BC. This method has the potential to fundamentally reverse the long-standing clinical challenge of endocrine resistance in HR+ patients [47, 115].

Additionally, this combined intervention concept exhibits a more extensive potential for applicability when combined with cyclin-dependent kinase 4/6 (CDK4/6) inhibitors. This combination not only more effectively blocks cancer cell proliferation cycles but also strategically employs ferroptosis inducers to precisely target residual cells that elude cycle arrest and exist in quiescent or hybrid p-EMT states [146]. The primary objective of this multivariate combination strategy is to directly address a critical clinical challenge—the complete eradication of MRD following treatment. Offering a highly promising new chemical biology pathway toward the ultimate cure of advanced BC, this strategy significantly lowers the chance of BC recurrence in the long run. Importantly, the “event-driven” degradation of EIK scaffolds (like LMTK3 and AXL) offers a special benefit in overcoming resistance caused by hypoxia. PROTACs have the ability to destroy the physical platforms that regulate droplet stability and lipid mobilization, in contrast to traditional inhibitors. These degraders can push resistant cells back into a state of high ferroptotic sensitivity by “unmasking” PUFAs from their protective droplet reservoirs, even in hypoxic MRD environments.

### Bridging the Physical and Translational Divide: A Rigorous Closed-Loop Approach from In Situ Probe Tracing to In Vivo Signal Pathway Validation

The severe physicochemical challenges that small-molecule therapeutics encounter within the solid TME must be addressed at the chemical engineering level. The effective diffusion coefficient of hydrophobic small-molecule drugs is substantially reduced by the dense connective tissue proliferation (desmoplasia) that is a characteristic of BC [110]. In particular, certain ferroptosis inducers that contain highly reactive covalent warheads (e.g., chloroacetamides) frequently confront issues such as poor stability and susceptibility of targeting groups to metabolic enzyme degradation during complex in vivo circulation. This poses a challenge for their ability to effectively penetrate minimal-residual disease (MRD) niches at high concentrations [147, 148]. Consequently, strategies must incorporate forward-thinking delivery chemistry in addition to affinity optimization. This encompasses the creation of “matrix-penetrating” prodrugs or clever nanosystems that are environmentally responsive (e.g., low pH/high glutathione). These methods guarantee the reproducibility of the “synthetic lethality” effect in the in vivo environment by optimizing pharmacokinetics (PK) and tissue distribution.

Future evaluation systems must expand their scope beyond in vitro biochemical experiments and concentrate on animal testing platforms in order to rigorously validate the efficacy of interventions [149]. The clinical translation of this novel paradigm is not supported by the sole reliance on traditional in vitro Western blot assays to confirm target kinase degradation [150]. The probe penetration efficacy within dense stroma is accurately reflected by patient-derived xenograft (PDX) models, which maximize the preservation of primary tumor molecular characteristics and stromal heterogeneity [151]. In the interim, immunocompetent syngeneic mouse models facilitate additional research into the adaptive remodeling of the anti-tumor immune microenvironment by interventions [152]. It is essential to systematically validate the tissue penetration kinetics, targeted persistence, and absolute advantage of these degraders in inducing massive ferroptosis within tumor parenchyma in highly reductive in situ tumorigenesis models that replicate dense stroma and immune microenvironments [153].

Future research paradigms should boldly establish a rigorous closed-loop system that spans “in situ probe tracing to in vivo biochemical validation” in order to truly cross this “translational gap.” Initially, directly apply bioorthogonal probes (such as terminal-alkyne-labeled Alk-AA) to mouse in situ tumor models or ex vivo fresh tissue sections. Visualize the spatial characteristics of drug-resistant clones that abnormally accumulate PUFAs within lesions that possess intact three-dimensional microenvironments by combining in situ click chemistry with tissue clearance and imaging.

Furthermore, standard tissue lysis and bulk biochemical assays are inadequate for accurately capturing the physiological relevance and complexity of tumors, as they obliterate essential spatial and microenvironmental context [154]. Rather, sophisticated spatially resolved technologies should be employed to validate targeted degradation and subsequent ferroptotic events in vivo. For example, mass spectrometry imaging (MSI) can precisely depict drug penetration and specific oxidized phospholipid profiles within the dense stroma and MRD compartments [155–157]. At the same time, spatial transcriptomics or multiplexed immunofluorescence (mIF) can assess the actual blockade of downstream core signaling pathways (e.g., PI3K/Akt or YAP/TAZ) and capture the adaptive remodeling of immune cell interactions, such as macrophage polarization, at single-cell resolution within the intact TME [154, 158]. The ‘EMT-ferroptosis’ synthetic lethal effect is validated under clinically pertinent, physiological conditions as a result of the integration of in situ probe tracing with high-dimensional spatial pathology [159].

### The Dynamic Evolution of Drug Resistance: Beware the “Second Wave” of Metabolic Remodeling

Translational medicine has faced a persistent challenge for a long time due to the extraordinary plasticity of cancer cells [18, 19]. It is crucial to maintain a high level of vigilance in the clinical application of interventions targeting the “EMT-ferroptosis” axis. An adaptive “compensatory shift” in metabolic dependence inside BC cells may be triggered by prolonged therapeutic pressure from particular ferroptosis-inducing drugs (FINs, such as GPX4 inhibitors) [102]. Rather than launching a de novo “second wave” of evolution, resistant clones dynamically redistribute their survival weight across extant antioxidant networks. Their actions redirect the primary metabolic burden from the targeted GPX4 axis to parallel, pre-sensitized systems, including the FSP1 or GTP cyclohydrolase 1 (GCH1)/Tetrahydrobiopterin (BH4) pathways [46, 160–162]. Undoubtedly, the dynamic reallocation of antioxidant dependency presents a significant challenge to single-target ferroptosis induction strategies, underscoring the profound metabolic plasticity of the p-EMT-state.

Future chemical biology research urgently necessitates an upgrade in intervention paradigms in order to effectively address this dynamically evolving resistance challenge and entirely restrict the escape routes of cancer cells. Next-generation “dual-target” or “multi-target” innovative small-molecule pharmaceuticals should be the primary focus of research efforts. This multidimensional chemical intervention network has the potential to fundamentally exhaust the metabolic compensation limits of BC cells by utilizing precise chemical probes and lead compounds to simultaneously block the GPX4 core pathway and synergistic antioxidant defense pathways (such as FSP1 or GCH1). This strategy preemptively eliminates any potential avenues for compensatory shifts. The objective of this method is to accomplish the complete and sustained suppression of drug-resistant clones within intricate evolutionary networks.

### Multimodal Liquid Biopsy: Redefining the “Clinical Navigation System” for MRD Eradication and Perioperative Intervention

The design of clinical trials for chemical biology pharmaceuticals is significantly influenced by the identification of reliable clinical biomarkers. Liquid biopsy has exhibited substantial potential in this translational process [115, 134]. However, it is essential to recognize that ctDNA-based applications now dominate the clinical landscape of liquid biopsy in BC. Lipid-based biomarkers are still a developing and mostly exploratory field, despite the enormous preclinical potential of lipidomics and exosomal lipid profiling in cancer biofluids. In particular, there is currently no clinical validation for an oxidized-lipid liquid biopsy technique specific to ferroptosis. We suggest that when analytical methods develop, researchers and doctors may eventually assess the severity of ferroptosis-induced reactions in real time, despite these present translational challenges.

However, the direct detection of free L-OOH or their degradation products (such as malondialdehyde, MDA or 4-hydroxynonenal, 4-HNE) in plasma is met with specificity challenges, as systemic inflammation or metabolic diseases may increase the background noise levels of these universal markers [134]. Future chemical biology strategies must evolve beyond the basic detection of aldehydes to identify “specific oxidized phospholipid fingerprints” in order to accurately track “tumor-specific” ferroptosis events within the intricate blood environment [115, 133].

Specific oxidized phosphatidylethanolamines that are enriched in tumor membranes, such as oxPE-C18:0/C20:4, can be identified as core characteristic peaks through high-resolution mass spectrometry, rather than generalized degradation fragments [133]. More importantly, in order to logically exclude non-tumor-derived interference, we suggest a “multi-modal liquid biopsy” strategy that spatiotemporally couples the dynamic changes of oxidized lipids with the release kinetics of circulating tumor DNA (ctDNA) [163]. We can confidently assert that the detected signals originate from BC cells undergoing ferroptosis, rather than systemic background noise, only when specific oxidized lipid peaks exhibit high temporal synchrony with ctDNA release from tumor-specific mutations (e.g.,*TP53* or *PIK3CA*) [164, 165].

The fundamental value of implementing this multimodal liquid biopsy system is not limited to early diagnosis. This involves the precise eradication of MRD and the provision of real-time “clinical navigation” for the perioperative management of BC [166, 167]. For an extended period, clinical practice has maintained a disproportionate dependence on conventional anatomical imaging evaluations for the dynamic monitoring of MRD. Nevertheless, drug-resistant clones have frequently already completed lethal systemic metastasis and metabolic remodeling by the time macroscopically visible or radiographically detectable recurrent lesions appear in coarse anatomical structures [168].

Conversely, a molecular-level “Ferroptosis Score” that surpasses conventional imaging resolution is achieved through multimodal liquid biopsy, which is based on “specific oxidized lipid fingerprint + ctDNA release kinetics“ [163]. In the aftermath of the primary tumor’s surgical resection, this dynamic surveillance provides critical guidance during adjuvant therapy. A clear warning to clinicians is provided when residual drug-resistant lesions are in a p-EMT-associated “dormant phase,” and conventional therapies have failed to effectively trigger ferroptosis, as evidenced by persistently elevated tumor-specific EMT vesicle signals in peripheral blood, which do not coincide with the release of lipid peroxidation products [18, 19, 169].

At this juncture, the “navigation system” can assist clinicians in making a decisive decision to alter intervention strategies, thereby precisely targeting the metabolic vulnerability window of MRD. For example, a persistently low “Ferroptosis Score” in the presence of high circulating EV-AXL or EV-PAK1 levels would logically trigger a switch from a conventional FIN to a specific PROTAC (e.g., an AXL-PROTAC or a PAK1-PROTAC) to dismantle the physical scaffold driving EMT and re-sensitize the MRD to ferroptosis [78]. This facilitates the opportune introduction of PROTACs that target scaffold kinases or potent ferroptosis inducers for eradication. In contrast, the successful induction of MRD into catastrophic ferroptosis is considered “direct molecular confirmation” when post-treatment blood samples demonstrate the synchronous, dramatic release of ctDNA in conjunction with specific oxidized phospholipid peaks [133]. The current passive situation where BC drug resistance and recurrence remain clinically elusive and unpredictable will be fundamentally altered by this forward-thinking vision of transforming chemical biology probe technology into a clinical dynamic monitoring “radar.” It offers a powerful molecular armament for the ultimate clinical cure following surgical intervention [134].

Additionally, EVs are non-invasive predictive markers that possess substantial translational value [103]. The detection of EVs in blood enriched with core EMT-driving kinases or specific lipid profiles provides a robust surrogate marker for determining whether tumor tissue has undergone EMT. Additionally, this indicator is essential for evaluating the high sensitivity of the tumor tissue to ferroptosis therapy [103, 105, 133, 170]. Ultimately, a diagnostic system that is constructed using these multidimensional biomarkers will provide clinicians with a significant advantage in the accurate stratification of BC patients. This guarantees that the most appropriate patient populations can legitimately benefit from chemical biology interventions that target the EMT-ferroptosis axis, thereby providing clear clinical guidance for progressing toward precision oncology.

## Conclusion

It is important to transcend the conventional binary perspective on EMT and concentrate on the specific metabolic vulnerabilities that are inherent to the p-EMT state in order to overcome treatment-resistant BC. The EMT program induces a unique “ferroptotic window” as a result of the profound lipidomic reprogramming and metabolic addiction that are emphasized in this Perspective. The precarious reliance on antioxidant defense systems to counterbalance the lethal accumulation of highly oxidizable polyunsaturated fatty acids is established by metastable p-EMT cells, which are actively “locked” in this vulnerable window by the scaffold networks of LMTK3 and AXL.

Our therapeutic paradigm needs to shift from traditional “occupancy-driven” inhibition to “event-driven” targeted protein degradation in order to fully take advantage of this weakness. The non-catalytic scaffolding networks of these hidden EIKs can be physically disassembled using chemical biology methods, including PROTACs. The pro-EMT cellular infrastructure is irrevocably destroyed by this physical eradication, which also compromises their compensating antioxidant defenses and sends resistant clones into catastrophic lipid peroxidation.

Three fundamental pillars are necessary for the successful clinical translation of this synthetic lethality strategy: first, stratifying patients according to p53 status to accurately predict their intrinsic ferroptosis sensitivity; second, using sophisticated PROTAC degraders to target hidden scaffolding super-hubs (e.g., LMTK3, PAK1) for the eradication of MRD; and third, putting in place a multimodal liquid biopsy system that links particular oxidized phospholipid signatures with ctDNA release kinetics to dynamically monitor this eradication in real-time.

In the end, chemical biology gives us both the “scalpel” to remove drug resistance at its molecular source and the “microscope” to map dynamic tumor progression. We can systematically eradicate MRD and provide a revolutionary road map to overcome BC dormancy by redefining the p-EMT state as a targetable metabolic reliance rather than an impregnable stronghold.

**Fig. 1:**
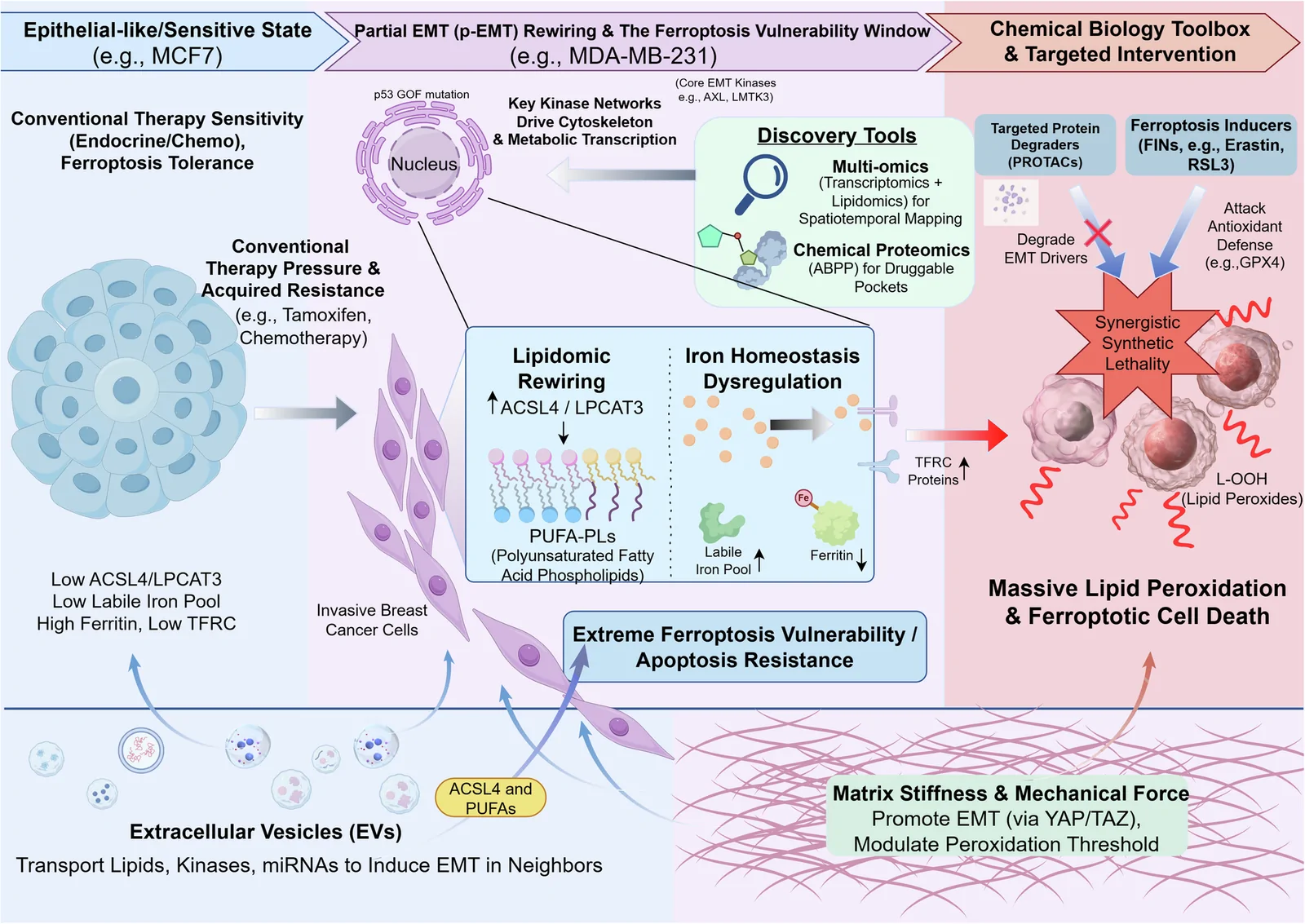
Unlocking the ferroptotic window: Targeting lipidomic rewiring and metabolic addiction in the EMT-driven resistance axis. Epithelial BC cells develop resistance by undertaking EMT and acquiring a mesenchymal phenotype in response to stress from conventional therapies (e.g., endocrine therapy, chemotherapy) [18, 19]. This transition is facilitated by a core EMT-induced kinase (EIK) network that integrates extracellular matrix signals to precisely modulate downstream transcription factors (e.g., SREBP1, Yes-associated protein/Transcriptional coactivator with PDZ-binding motif (YAP/TAZ)), to directly drive the enrichment of membrane polyunsaturated fatty acid phospholipids (PUFA-PLs) [68, 171]. A unique ferroptosis susceptibility window is created by this compositional alteration, which is powered by the overexpression of enzymes like Acyl-CoA Synthetase Long-Chain Family Member 4 (ACSL4) and Lysophosphatidylcholine Acyltransferase 3 (LPCAT3) [19, 69]. Additionally, an extended labile iron pool is created by changed iron transport (increased transferrin receptor (TFRC)) [47–49]. Researchers have utilized activity-based proteomics (ABPP) technologies and multi-omics integration to precisely identify concealed druggable regions within EIK networks, thereby facilitating the development of innovative small-molecule inhibitors [118, 172]. In the presence of ferroptosis inducers (FINs, such as Erastin and RSL3), these inhibitors result in potent synergistic lethal effects by inhibiting survival drivers and enhancing metabolic dysregulation, thereby initiating catastrophic lipid peroxidation and ferroptosis [18, 50]. Additionally, the tumor microenvironment (TME) exerts a significant influence on this axis, which includes signal transmission through extracellular vesicles (EVs) and mechanical signal transduction from matrix stiffness [68, 102].
